# Association between red blood cell distribution width and non-valvular atrial fibrillation in hemodialysis patients: a single-center Chinese population study

**DOI:** 10.1080/0886022X.2021.2019588

**Published:** 2022-02-14

**Authors:** Tao Zhang, Zhengjie Zhu, Hongtao Yang, Shili Cao, Jing Li, Qingmiao Shao

**Affiliations:** a Department of Nephrology, First Teaching Hospital of Tianjin University of Traditional Chinese Medicine, National Clinical Research Center for Chinese Medicine Acupuncture and Moxibustion, Tianjin, People’s Republic of China; b Department of Nephrology, First Central Hospital of Tianjin, Tianjin, People’s Republic of China; c Tianjin Key Laboratory of Ionic-Molecular Function of Cardiovascular disease, Department of Cardiology, Tianjin Institute of Cardiology, the Second Hospital of Tianjin Medical University, Tianjin, People’s Republic of China

**Keywords:** Red blood cell distribution width, non-valvular atrial fibrillation, hemodialysis

## Abstract

**Background:**

Red blood cell distribution width (RDW) has emerged as a prognostic marker of atrial fibrillation (AF) in various clinical settings. However, the relationship by which RDW was linked to AF in hemodialysis (HD) patients was not clear. We sought to reveal the relationship between RDW and AF occurrence in HD patients.

**Methods:**

We enrolled 170 consecutive maintenance HD patients, including 86 AF patients and 84 non-AF patients. All participants’ medical history and detailed clinical workup were recorded before the first dialysis session of the week. Electrocardiography, laboratory and transthoracic echocardiography examination indices were compared between the AF group and non-AF group. Multivariable logistic regression analysis was performed to identify the independent predictors of AF occurrence in HD patients.

**Results:**

There were all paroxysmal AF patients in AF group. Compared to the non-AF group, patients with AF group had a significantly older age (61.0 ± 1.48 vs. 49.71 ± 1.79, *p* < 0.001), lower BMI (24.3 ± 4.11 vs. 25.8 ± 3.87, *p* < 0.05), higher RDW (15.10 ± 0.96 vs. 14.26 ± 0.82, *p* < 0.001) and larger LAD (39.87 ± 3.66 vs. 37.68 ± 5.08, *p* < 0.05). Multivariable logistic regression analyses demonstrated that values of age (OR: 1.030, 95%CI: 1.004-1.057, per one- year increase), BMI (OR: 0.863, 95%CI: 0.782–0.952, per 1 kg/m^2^ increase), RDW (OR: 2.917, 95%CI: 1.805–4.715, per 1% increase) and LAD (OR: 1.097, 95%CI: 1.004–1.199, per 1 mm increase) were independently associated with AF occurrence (*p* < 0.05, respectively). The best cutoff value of RDW to predict AF occurrence was 14.65% with a sensitivity of 68.6% and a specificity of 72.6%.

**Conclusions:**

The increased RDW was significantly associated with the paroxysmal AF occurrence in HD patients.

## Introduction

Atrial fibrillation (AF) is one of the most common clinical arrhythmia and is associated with morbidity and cardiovascular mortality [1]. Patients with chronic kidney disease (CKD) are at elevated risk of AF occurrence, especially in those undergoing maintenance hemodialysis [2,3]. It is well known that inflammation has been associated with occurrence and development of AF and CKD [4]. In addition to the conventional risk factors for AF, such as age, obesity, hypertension, and diabetes mellitus (DM), are found to correlate with AF occurrence in HD patients [5,6]. AF and CKD frequently coexist, amplifying the risk of cardiovascular events and mortality [7]. In view of cardiovascular mortality of hemodialysis, early warning and decision-making are needed. Therefore, it is necessary to find a more promising and inexpensive value of novel biomarker of AF occurrence in HD population.

Red blood cell distribution width (RDW) is a quantitative index that reflects the volumetric heterogeneity of erythrocyte in peripheral blood. Elevated RDW is not only associated with enhancive destruction and ineffective production of erythrocyte, but also indicates inflammation and malnutrition status [8,9]. Meanwhile, RDW, as an indicator of inflammation [9], has been regarded as a novel and independent prognosis predictor of in various clinical settings, including acute and chronic heart failure, [10,11] stroke, [12] kidney transplant [13] and CKD [14–16]. Most notably, a growing number of studies evaluated that elevated RDW has been proven to be related to increased incidence of AF in patients with cardiac procedure or surgery [17,18], hypertension, [19] sick sinus syndrome, [20] percutaneous coronary intervention (PCI) [21]. Moreover, Jurin et al. studied that RDW was not only associated with AF incidence, but also was independently associated with AF progression. Patients with RDW level ≤ 14.5% are most probably the best candidates for rhythm control strategies [22]. Existing studies have highlighted that RDW was associated with poor outcomes in end-stage renal disease (ESRD) patients [23,24]. Notably, Mo et al [24] found that increased RDW was an independent risk factor for cerebral infarction in HD patients. However, the relationship between RDW and AF occurrence in HD patients is unclear. Therefore, the purpose of this study was to evaluate the association between RDW and non-valvular AF occurrence in maintenance HD patients.

## Methods

### Patient enrollment

From 1 March 2017 to 1 September 2020, we recruited consecutive maintenance HD patients who were admitted to our department of nephrology in the retrospective cross-sectional (snapshot) study. All patients enrolled were aged >18 years and had a dialysis vintage >3 months (three times per week for 4-h session). Exclusion criteria were showed as follows: acute and chronic heart failure, valve diseases, acute myocardial infarction, hyperthyroidism or hypothyroidism, hepatic insufficiency, autoimmune diseases, malignant tumor and acute inflammatory or infectious diseases. In addition, the patients who gave the red blood cells by intravenous infusion for nearly three months were also excluded from the study. Then, the remaining patients were divided into two groups according to the presence of paroxysmal AF: AF group and non-AF group. The diagnosis of paroxysmal AF based on only 12-lead electrocardiogram (ECG) or one 24-h ECG analysis acquired at baseline. AF was classified as paroxysmal based on clinical assessment and 12-lead electrocardiogram (ECG) or 24-h Holter recording according to European Society of Cardiology Guideline [25],

### Study protocol

All clinical data included medical history, 12-lead electrocardiography (ECG), and transthoracic echocardiography were regularly collected once they were on admission. Fasting venous blood samples were collected from the antecubital vein of the participants from 6:00 a.m. to 6:30 a.m. on the second day of admission before the start of the first dialysis session of the week. Complete blood count (CBC) was tested using semiconductor laser flow cytometry and nucleic acid staining method by an (SYSMEX XS-500i, Chuo-Ku, Kobe, Hyogo, Japan) automatic blood analyzer for total white blood cell (WBC), red blood cell (RBC), hemoglobin (Hgb), and RDW. Blood biochemical indexes such as serum creatinine (Cr), urea nitrogen (BUN), and serum potassium (K) were measured after overnight 8 h fasting conducted by an ARCHITECT ci16200 analyzer (Abbott, USA).

A transthoracic echocardiographic examination was performed in all patients using the LOGIQ E9 system equipped with new Agile ultrasonic platform (GE Medical Systems, Milwaukee, WI, USA). Echocardiographic parameters were obtained using two-dimensional imaging in the parasternal long-axis, apical four-chamber and two-chamber views during five consecutive cardiac cycles. Left atrial anteroposterior diameter (LAD) was measured during left ventricular end systolic, while interventricular septal thickness (IVST), left ventricular posterior wall thickness (LVPWT) and left ventricular end-diastolic diameter (LVEDD) were measured during left ventricular end diastolic. Left-ventricular ejection fraction (LVEF) was analyzed using Simpson’s biplane formula. Operation and measurement were performed by the same ultrasound doctor who was blind to participants' clinical information.

### Statistical analysis

The Kolmogorov–Smirnov test was used to testify the normality of included variables and a *P* value >0.05 was defined as normally distributed data. Continuous variables were presented as means ± SD, and median (interquartile range) if necessary. Categorical variables were reported as counts (percentage). The difference between the categorical variables was compared Chi square test. The Student’s *t*-test was used to compare the difference between two normal distribution continuous variables, whereas Mann–Whitney U-test was used to compared the difference between non-normal distribution continuous variables. We used multivariable logistic regression analysis to identify the independent predictors of AF occurrence in HD patients. The variables that differed between the two groups were substituted into the multivariate logistic regression equation. ROC curves were generated to determine the cutoff value, the sensitivity, and specificity of RDW to predict AF occurrence in HD patients. Statistical analysis was carried out using SPSS Ver. 22 Software (SPSS Inc., Chicago, IL, USA). A *P* value <0.05 was considered to be statistically significant.

## Results

### Clinical and echocardiographic characteristics

**Figure 1.**
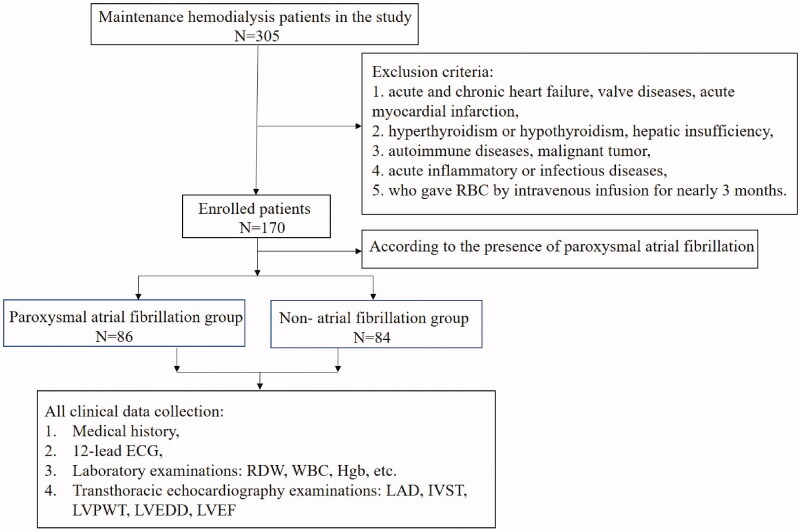
The flowchart of the selected population in this study.

Figure 1 was the flowchart of the selected population in this study. Clinical and echocardiographic characteristics of study population were shown in Table 1. Patients with the AF and the non-AF group had a similar distribution in previous history, such as DM, hypertension, stroke, and medications. While patients with AF group had higher proportion of history of DM and stroke, and β-blocker use when compared to the non-AF patient group, there was no statistically significant difference. The patients with the AF group had a significantly older age (61.0 ± 1.48 vs. 49.71 ± 1.79, *p* < 0.001), lower BMI (24.3 ± 4.11 vs. 25.8 ± 3.87, *p* < 0.05), and larger LAD (39.87 ± 3.66 vs. 37.68 ± 5.08, *p* < 0.05) than those of in the non-AF group.

**Table 1.** Baseline clinical and echocardiographic characteristics of study population.

	Non-AF (*n* = 84)	AF (*n* = 86)	*p* Value
Age, years	49.71 ± 1.79	61.0 ± 1.48	<0.001
Sex, female (*n*, %)	34 (40.5)	42 (48.8)	0.285
Diabetes mellitus (*n*, %)	40 (47.6)	50 (58.6)	0.219
Hypertension (*n*, %)	84 (100)	86 (100)	
BMI (kg/m^2^)	25.8 ± 3.87	24.3 ± 4.11	0.015
Stroke (*n*, %)	20 (23.8)	28 (32.6)	0.235
Smoking habit (*n*, %)	28 (33.3)	24 (27.9)	0.507
Drinking habit (*n*, %)	17 (23.8)	16 (18.6)	0.456
Number of dialysis (week)	3.00 ± 0.00	3.02 ± 0.150	0.162
Time of dialysis (mon)	27.81 ± 5.02	25.26 ± 3.70	0.681
Single pool-Kt/V	1.28 ± 0.15	1.27 ± 0.20	0.829
CHA_2_DS_2_-VASc score		3.42 ± 1.20	
LADS score	1.50 ± 0.83	1.65 ± 0.76	0.218
HATCH score	1.73 ± 0.96	2.01 ± 0.90	0.003
Previous medication (*n*, %)			
ACEI/ARB	36 (42.9)	36 (41.9)	1.000
Beta-blocker	52 (61.9)	60 (69.8)	0.332
Calcium channel blocker	76 (90.5)	72 (83.7)	0.254
Aspirin	17 (20.2)	18 (20.9)	1.000
Statin	12 (14.3)	20 (23.3)	0.170
Calcium	29 (34.5)	26 (30.2)	0.624
Dephosphorization agent	37 (44.0)	34 (39.5)	0.641
Iron	30 (35.7)	28 (32.6)	0.747
Erythropoietin	84 (100)	84 (97.7)	0.497
VKA or Rivaroxaban	3 (3.6)	12 (14.0)	0.028
Echocardiographic indicator			
LAD (mm)	37.68 ± 5.08	39.87 ± 3.66	0.001
IVST (mm)	10.32 ± 1.43	10.54 ± 1.44	0.307
LVPWT (mm)	9.36 ± 1.26	9.59 ± 2.13	0.387
LVEDD (mm)	50.33 ± 6.96	50.07 ± 5.92	0.790
LVEF (%)	60.81 ± 9.72	58.44 ± 10.53	0.130

BMI: body mass index; ACEI: angiotensin-converting enzyme inhibitor; ARB: angiotensin receptor blocking agents; LAD: left atrial diameter; IVST: interventricular septal thickness; LVPWT: left ventricular posterior wall thickness: LVEDD: left ventricular end-diastolic diameter; LVEF: Left ventricular ejection fraction.

### Laboratory parameters

Table 2 showed baseline laboratory characteristics of enrolled participants. The RDW level was significantly higher in the AF group than in the non-AF group (15.10 ± 0.96 vs. 14.26 ± 0.82, *p* < 0.001). The levels of Hgb, C-reactive protein (CRP), albumin (ALB) and Cr were found no significant differences between the two groups.

**Table 2.** Laboratory parameters of study population.

	Non-AF (*n* = 84)	AF (*n* = 86)	*p* Value
White blood cell Count (10*9/L)	6.62 ± 1.68	6.36 ± 1.74	0.319
Red blood cell Count (10*12/L)	3.71 ± 0.67	3.53 ± 0.68	0.081
Hemoglobin (g/L)	108.14 ± 19.14	102.63 ± 18.49	0.058
Red blood cell distribution width (%)	14.26 ± 0.82	15.10 ± 0.96	<0.001
Serum creatinine (µmol/L)	691.93 ± 30.8	693.45 ± 20.47	0.967
Urea nitrogen (mmol/L)	20.26 ± 9.75	20.96 ± 7.48	0.600
Uric acid (µmol/L)	371.79 ± 14.4	384.40 ± 8.41	0.448
Serum potassium (mmol/L)	4.70 ± 0.62	4.51 ± 0.71	0.059
Carbon dioxide binding capacity (mmol/L)	21.15 ± 0.43	21.37 ± 0.34	0.674
Fasting blood-glucose (mmol/L)	6.67 ± 0.83	6.78 ± 0.79	0.381
C-reactive protein (mg/L)	20.99 ± 1.56	20.31 ± 1.63	0.764
Triglyceride (mmol/L)	1.77 ± 0.14	2.08 ± 0.21	0.224
Total cholesterol (mmol/L)	4.36 ± 1.63	4.66 ± 1.30	0.193
Low density lipoprotein cholesterol (mmol/L)	2.34 ± 0.14	2.64 ± 0.11	0.187
Serum calcium(mmol/L)	2.19 ± 0.17	2.18 ± 0.23	0.665
Serum phosphorus(mmol/L)	1.83 ± 0.67	1.69 ± 0.47	0.099
Total parathyroid hormone (pg/mL)	398.10 ± 23.76	405.30 ± 42.61	0.884
Serum magnesium (mmol/L)	1.04 ± 0.14	0.97 ± 0.25	0.052
Plasma albumin (g/L)	36.10 ± 0.94	35.52 ± 0.56	0.591
Aspartate aminotransferase (U/L)	13.40 ± 0.46	12.73 ± 0.67	0.408
Alanine aminotransferase (U/L)	13.81 ± 0.72	11.46 ± 1.27	0.110
Homocysteine cycle enzyme (µmol/L)	30.88 ± 2.55	30.70 ± 2.27	0.957
Serum iron concentration (µmol/L)	10.09 ± 0.65	10.86 ± 0.66	0.405
Total iron binding force (µmol/L)	45.28 ± 9.12	44.50 ± 10.18	0.600
Iron transferrin saturation (%)	24.19 ± 1.86	25.47 ± 1.69	0.613

### Multivariate logistic regression analyses of AF occurrence in HD patients

In the multivariate logistic regression analysis, the result demonstrated that the values of age (OR: 1.030, 95%CI: 1.004–1.057), BMI (OR: 0.863, 95%CI: 0.782–0.952), RDW (OR: 2.917, 95%CI: 1.805–4.715) and LAD (OR: 1.097, 95%CI: 1.004–1.199) were independently risk factors of AF occurrence in HD patients (*p* < 0.05, respectively) after adjusted gender, number of dialysis, Hgb, serum potassium and CRP in Table 3. McFadden’s pseudo-R-square for RDW in the logistic regression model was 0.396.

**Table 3.** Multivariate logistic regression analysis on predictors of paroxysmal AF in the HD population.

	B	SE	Wald	*p* Value	OR	95% CI	
Age, per 1 year increase	0.030	0.013	5.074	0.024	1.030	1.004	−1.057
BMI, per 1kg/m^2^increase	−0.148	0.050	8.637	0.003	0.863	0.782	−0.952
RDW, per 1% increase	1.071	0.245	19.091	<0.001	2.917	1.805	−4.715
LAD, per 1mm increase	0.093	0.045	4.160	0.041	1.097	1.004	−1.199
HATCH score, per 1 score increase	0.295	0.227	1.683	0.195	1.343	0.860	−2.095

BMI: body mass index; RDW: red blood cell distribution width; LAD: left atrial diameter; HATCH score represented hypertension, age, ischemic events, chronic obstructive pulmonary disease, and heart failure.

### Predictive value of RDW and LAD for AF occurrence in HD patients

**Figure 2.**
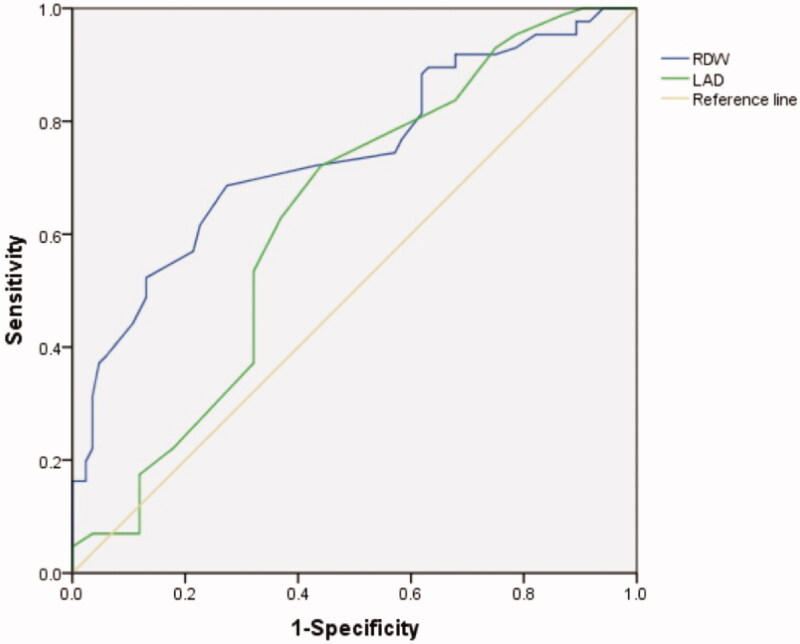
Receiver-operating characteristic (ROC) curves for RDW and LAD values in prediction of AF in HD patients.

The AUC under the ROC curve of RDW and LAD to predict AF occurrence in HD patients was 0.740 (95%CI: 0.666-0.814, *p* < 0.001) and 0.632 (95%CI: 0.547-0.717, *p* < 0.05) respectively. The best cutoff value of RDW to predict AF occurrence in HD patients was 14.65%. A RDW value higher than 14.65% has a sensitivity of 68.6% and a specificity of 72.6% (Figure 2). ROC analysis revealed that a LAD measurement higher than 38.5 predicted AF occurrence with a sensitivity of 72.1% and a specificity of 56% (Figure 2).

## Discussion

This single-center retrospective cross-sectional study evaluated the association between RDW and AF occurrence in 170 HD patients. Our study showed higher RDW in patients with AF than that in non-AF patients, and RDW was independently associated with AF occurrence in HD patients. In addition, ROC analysis revealed that the best cutoff value of RDW to predict AF occurrence was 14.65% with a sensitivity of 68.6% and a specificity of 72.6%.

AF is one of common causes of increased adverse cardiovascular events and mortality, and has placed great burdens on the global healthcare system [26]. It is widely known that AF occurrence is related to age, obesity, DM, hypertension, and renal dysfunction [27,28]. Patients with reduced renal failure are at elevated risk of AF, especially in those undergoing hemodialysis [29,30]. Epidemiologic studies suggested that the incidence of AF was higher in ESRD patients with dialysis (27%) compared with non-dialysis-dependent CKD patients (18-21%) and elderly people without CKD (6%) [30]. Moreover, the changes in the stage of ESRD with dialysis is usually complex, during which fluid overload, malnutrition, accumulation of uremic toxins and waste products, and electrolyte imbalance are common. These changes could lead to exacerbate inflammation and simultaneously increased disproportionately higher cardiovascular mortality [31]. A growing body of evidence demonstrated that inflammation and oxidative stress have been implicated in the pathophysiology of atrial remodeling in AF. AF and CKD are both progressive diseases, which accompanying by similar or common risk factors, [6,7,32–34] jointly amplifying the risk of morbidity and cardiovascular mortality.

RDW is traditionally simple and cost-effective to measure for differential diagnosis of anemia in clinical practice. Some studies suggested that high levels of RDW reflected an activated inflammatory state. Specifically, inflammation and oxidative stress may inhibit erythrocyte maturation. Other studies have also demonstrated an association between increased RDW and adverse perinatal outcomes of various clinical diseases, including acute and chronic heart failure, stroke, kidney transplant and CKD [10–16]. Lately, several published works explored the potential relationship between AF occurrence and RDW in patients with cardiac procedure or surgery, hypertension, sick sinus syndrome, percutaneous coronary intervention (PCI) [17–21]. We suggested that RDW may be a supplement to dialysis markers available currently in the study, which do not represent AF. Considering that complete blood count is the simplest and most cost-effective routine measurement in clinical practice, we think that RDW is a promising marker for AF in HD patients.

The exact mechanisms underlying the association between RDW and AF occurrence are not clear, and may be explained by several factors. First, inflammatory factors and oxidative stress are important mechanisms causing AF and adverse outcomes in CKD patients. Also, inflammation inhibits bone marrow function and iron metabolism [35–37]. Pro-inflammatory cytokines reduce the sensitivity of red bone marrow stem cells to erythropoietin and inhibit erythropoietin-induced erythrocyte maturation and proliferation, leading to more immature red blood cells released into the peripheral blood circulation, which finally result in a rise in RDW [38]. Meanwhile, oxidative stress not only decreases renal secretion of erythropoietin, but also leads to ineffective erythropoiesis by inhibiting RBC maturation, and thereby promoting anisocytosis [39]. An increasing number of studies have suggested that inflammation and oxidative stress contributed to atrial remodeling and AF occurrence, development and progression [5]. Compared with the general population, patients receiving HD have higher levels of inflammation and oxidative stress due to various factors including blood touching with the dialysis membrane, microbial contamination of the dialysate, and decreased activity of the glutathione system [7]. Second, it is generally that malnutrition is more prevalent among CKD patients, particularly those receiving long-term dialysis. It has been well documented that high RDW levels have been significantly associated with malnutrition. Our study found that patients with AF group had a higher RDW, while a lower BMI than the non-AF group. Third, chronic hypoxia or endothelial dysfunction may be also related to the mechanism [38,40]. Finally, elevated RDW reflects impaired iron metabolism. Observed changes in RDW indicated hemodynamic overload changes, which may play a significant role in the mechanism of arrhythmia.

Some limitations should be considered in this study. Firstly, this is a retrospective study that only included patients from a single-center and the sample size is relatively small. The number of maintenance HD patients in our center was 305. A total of 135 dialysis patients were excluded based on the exclusion criteria, and most of these patients had no history of AF, so the incidence of AF is 30% in our dialysis institution. However, the high prevalence of paroxysmal AF in HD patients from our center may be related to the high morbidity of coronary heart disease, hypertension, sodium and water retention, and electrolyte disorder. Secondly, in view of the observational studies, we cannot determine the cause-and-effect relationship between RDW and AF. Thirdly, AF screening was based on clinical assessment and 12-lead electrocardiogram (ECG) or 24-h Holter recording, which may miss asymptomatic AF and paroxysmal AF patients. Fourthly, we also cannot completely rule out the multiple impacts of complex conditions on RDW, such as dialysis membrane, anticoagulants, and EPO resistance in HD patients. Hgb was found no significant difference between the AF and non-AF groups. Moreover, the result of multivariable analyses demonstrated that the values of RDW were independently risk factors for AF occurrence after adjusted gender, numbers of dialysis, Hgb, K and CRP. Therefore, Hgb has no effect on RDW being an independent factor in AF occurrence in the study. However, the cutoff RDW value may be deviations. Clinical evidences have shown that the value of RDW was closely related to degree of kidney function, malnutrition and inflammation. Fifthly, we do not have data on AF burden and other future untoward events. Despite these limitations, our study may help to distinguish high-risk of AF occurrence in HD patients. Finally, the large RDW confidence interval in this study may be caused by the small sample size or the relatively dispersed values among samples. It needs large multi-center prospective trials to further verification.

## Conclusions

In conclusion, the results of this study demonstrated that RDW values has statistically significant correlation with the AF occurrence in maintenance HD patients. Moreover, elevated RDW levels may be independently associated with the paroxysmal AF occurrence in HD patients after multivariable analyses. RDW is an inexpensively and promising marker of AF occurrence in HD patients.
